# Williamson magneto nanofluid flow over partially slip and convective cylinder with thermal radiation and variable conductivity

**DOI:** 10.1038/s41598-022-16268-2

**Published:** 2022-07-26

**Authors:** M. Bilal, Imran Siddique, Andrzej Borawski, A. Raza, M. Nadeem, Mohammed Sallah

**Affiliations:** 1 Department of Mathematics, University of Chenab, Gujrat, 50700 Pakistan; 2 Department of Mathematics, University of Management and Technology, Lahore, 54770 Pakistan; 3 Faculty of Mechanical Engineering, Bialystok University of Technology, 45C Wiejska Str., 15-351 Bialystok, Poland; 4 Applied Mathematical Physics Research Group, Physics Department, Faculty of Science, Mansoura University, Mansoura, 35516 Egypt; 5 Higher Institute of Engineering and Technology, New Damietta, Egypt

**Keywords:** Mathematics and computing, Nanoscience and technology, Physics

## Abstract

This article is concerned with the study of MHD non-Newtonian nanofluid flow over a stretching/shrinking cylinder along with thermal radiation effects. Two-component slip mechanism models, namely Brownian motion and thermophoresis of nanofluid for the mass and energy transportation, developed by Buongiorno, are used. Convective heat transfer and nonuniform magnetic field are retained for the expanding/contracting cylinder. Variable thermal conductivity and heat generation effects along with slip boundary conditions are utilized over the cylinder surface. By utilizing the similarity transformation, these governing partial differential equations are converted into nonlinear ordinary differential equations (ODEs). To obtain numerical results, these ODE’S are solved by the shooting method using MATLAB software. The impact of different parameters like variable thermal conductivity, radiation parameter, magnetic parameter, Prandtl number, Brownian motion parameter, the magnetic parameter, Weissenberg number, the viscosity ratio parameter and mass transfer parameter, on the velocity, temperature and concentration is discussed graphically. Further, the Sherwood number, Nusselt number, the skin friction coefficient are also discussed through figures. It is noted through analysis that the speed of the nanofluid reduces for the higher Weissenberg number and expanding cylinder. For the contracting cylinder, i.e., for the negative unsteadiness parameter, the velocity increases.

## Introduction

Fluid dynamics play an important role in many industrial processes, chemical engineering, biomedical field, and advanced technologies, especially, in nanotechnology. Many researchers are attempting to explain the integrity of fluid dynamics in a variety of practical applications in order to better understand its rheology. Engineers and scientists highlighted especially the non-Newtonian fluids as a key focus of their research. Many industrial liquids, such as solutions, certain fuels, paper materials, paints, cosmetics, slurries, oils, and polycrystal melts, have non-Newtonian fluid properties. It is an established fact that a single fluid expression cannot express all of the characteristics of all non-Newtonian fluids. Numerous previous literature reviews have revealed that non-Newtonian fluids can be described using various constitutive expressions. Several models have been developed to investigate pseudo-plastic (shear thinning) fluids, including the Ellis model, Cross model, Carreau model, power-law models, and so on. Williamson in his theory of Pseudoplastic fluid has elaborated the worth of fluid dynamics. It is very important due to its practical implementation in various industries like biological sciences, geophysics, petroleum, chemical industries, and so on. The pioneering study has been done by Sakiadis^1^ to examine the properties of the fluid flowing on the linearly stretched surface. Similar solutions were obtained by Crane^2^ while studying the fluid stream over the stretching sheet. He also discussed the closed-form of the exponential solution of the same problem. Gupta and Gupta^3^ formulated an expression for the heat and mass transmission rate with suction and blowing process together, over the stretched sheet. A variable heat flux on the stretched surface was studied by Elbashbeshy^4^. The radiation effect on the unsteady stretching sheet was inspected by Aziz El-Aziz^5^. Thermal radiation’s effect with porous medium on a vertically stretched sheet was investigated by Mukhopadyay^6^. A numerical study was conducted by Shateyi and Motsa^7^ to examine the mass and heat transfer rates on the plane sheet. The effect of MHD and thermal radiation on a permeable convective steep sheet with Dufour and Soret reactions for the mass and heat transfer purpose was studied by Aziz El-Aziz^8^. The above-mentioned work was further extended by Hady et al.^9^ with a viscous fluid flow over the nonlinear stretching sheet using nanofluid. The MHD impact on viscous fluid flowing on the linearly stretched sheet with constant density was examined by Pavlov^10^. The second law of thermodynamics is applied by Bianco et al.^11^ to optimize the entropy generation for the water-$\text{Al}_{2}\text{O}_{3}$ nanofluid flowing within the tube. They show how the entropy within the tube changes with the change of concentration, dimension, and inlet condition of the particle. Different fluid models over the linear and exponential stretching sheets were investigated by Nadeem et al.^12^. These fluid models have lots of applications in engineering, physics, and chemistry process. To make the refined quality of copper wires, the classical applications may include the cooling of electromagnetic fluid. Elbashbeshy and Bazid^13^ analyzed the time-dependent mass and heat transfer of suction and blowing processes. Noreen et al.^14^ studied analytically the Williamson nanofluid flow in an asymmetric channel. Imad et al.^15^ has used the analytical approach to discuss the chemical reaction impact through the cone and plate for the Williamson nanofluid. Ijaz et al.^16^ have studied the variable viscosity of Williamson nanofluid by using the entropy optimization with Joule heating and chemical reaction. For the Williamson nanofluid, Sami et al.^17^ used an oscillatory stretching sheet to investigate the influence of magnetic field on the heat and fluid flow.

The study of nanofluids has gotten a lot of attention in the present period of science and technology because of its wide range of applications in almost all sectors of science. Nanofluids are used in a variety of fields, including medical suspension, sterilization, aerospace, tribology, electronic component cooling, natural convection heat transfer, and a variety of other heat transfer applications such as heat exchangers, micro-channels, heat pipes, tubular heat exchangers, and so on. Nanofluids have shown improved thermo-physical properties such as convective heat transfer coefficients, thermal conductivity, thermal diffusivity, and viscosity when compared to base liquids in numerous studies. Nanofluids are basically the base fluid such as water, oil, and ethylene glycol with the suspension of nanoparticles. Over the past few decades, the quest to increase the efficiency of the equipment that can transfer heat has been accelerated. The concept of nanofluids was first introduced by Choi^18^. The suspension of the nanoparticles in the base fluids thus result an increase of thermal conductivity of the nanofluids which was explained by Buongiorno^19^ by formulating a model which took into account the Brownian movement and thermophoresis of the particles. Various active and passive methods have been devised for more efficient and consolidated heat transfer^20^. Experiments carried out by Kang et al.^21^ to prove the accuracy of Choi’s result. Ganji and Hatami^22^ analyzed the *Cu*-water nanofluid that flows between the two plates that are squeezing, by using DTM-Pade and NUM methods. Volder et al.^23^ investigated thoroughly and converted the vertically aligned CNTs filaments into the complex three-dimensional micro-architectures using the capillaries structure. Ghadikol et al.^24^ discussed the boundary layer flow of the micro-polar dusty fluids with $\mathrm{TiO}_{2}$ as the nanoparticles in porous medium while taking into account the magnetic field and thermal radiations. Akbar and Nadeem^25^ conducted the studies in which they viewed the impact of nanofluid in the endoscope. They also studied the characteristics of the nanofluids concerned with thermal conductivity. The treatment of the tumor is carried out by means of injecting the magnetic nanoparticles into the tumor affected part and subsequently heating them for curing it, was discussed by Landeghem et al.^26^. Some further study on nanofluids latest development can be noticed in Ref.^27–42^.

There has been a growing interest in researching magnetohydrodynamic (MHD) flow characteristics because of the impact of magnetic fields on flow management and the performance of various systems via electrically conducting liquids such as liquid metals, water mixed with a little acid, and so on. The scientific study of how a magnetic field influences a liquid is known as magnetohydrodynamics. MHD tube flows, MHD flow management in atomic reactors, biomedicine, medication delivery, cancer therapy, magneto-optical wavelength filters, and many other industries use MHD flows. The effects of cylindrical capillary radius on the electric-kinetic flow through a narrow tube were studies by Rice and Whitehead^43^ in 1965. A few years later, Sorensen and Koefoed^44^ studied the electro-kinetic effects in a charged capillary tube. The coefficients for electro-kinetics of the electrolyte solution filled in a narrow tube with surface charge were determined. The heat radiation influence of a persistent, viscous, incompressible water-based MHD nanofluid flow between two stretchy or shrinkable boundaries was investigated by Dogonchi and Ganji^45^. Padam and Kumar^46^ studied mass transfer and heat flow in a two-dimensional magnetohydrodynamic slip flow of an incompressible, electrically conducting, viscous, and steady flow of alumina water nanofluid in the presence of a magnetic field.

The physical properties of non-Newtonian Williamson fluid flow, as well as heat transmission in the presence of suspended nanoparticles is the main novelty of this article. On a cylinder, we attempted the erratic flow of magneto nanofluid. The flow is taken over the convective cylinder by using slip condition, variable thermal conductivity, and thermal radiation. The well established partial differential equations are converted into the ordinary differential equation by making the use of similarity transformation. A shooting method is utilized to solve the system of ODEs. Different graphs are drawn by using different parameters and discuss them physically. Nusselt number, Sherwood number are also calculated and discussed. In the end, we conclude the entire work. This kind of study can be useful in extrusion of polymer sheet, pipe industry, emulsion coated sheet, annealing, thinning of copper wire and so on.

## Mathematical formulation

**Figure 1:**
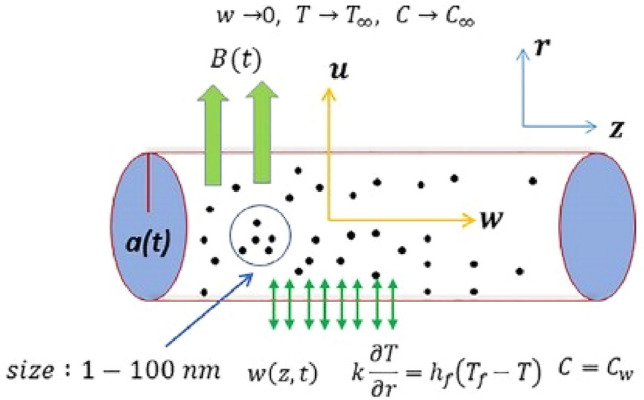
Flow over the stretching cylinder.

The diagrammatical representation of the expanding and contracting cylinder is given in Fig. 1. The major concern of this research is to study the heat transfer and its flow in non-Newtonian fluid with the suspension of nanoparticles that are allowed to pass over the convective boundary condition on an expanding/contracting cylinder.

Following the suppositions that are the subject of the study;

- Two-dimensional incompressible flow of the non-Newtonian fluid which is time-dependent.
- Buongiorno’s mathematical model for nanofluids is used.
- Viscous effects are much greater than the inertial effects.
- A slip condition is also applied on the boundary.
- Convective boundary conditions influence the heat transmission.
- A changeable magnetic field $H(t) =\frac{H_{0}}{1-B t}$ is applied perpendicularly where $H_{0}$ is its strength.
- A variable time dependent radius of the cylinder $a(t) =a_{0}\sqrt{1-B t}$ is taken
- Thermal radiation *Rd* and thermal conductivity *K* effects are used which are non-linear.
- Williamson fluid model with non-Newtonian characteristics.

Cylindrical coordinates (*r*, *z*) are selected which are mainly concerned with the direction of the flow. In this study, we suppose that there has some variation in the diameter of the cylinder as a function of time. If the value of the *B* is positive then over time the diameter of the cylinder will reduce, i.e., contracting cylinder. The negative value of the *B* indicates that the diameter of the cylinder is increasing with time, i.e., expanding. According to the above-mentioned hypotheses, the major equations in vector notation for the unstable Williamson fluids are:

1 $$\begin{aligned}&\nabla \cdot {\textbf {V}}=0, \end{aligned}$$

2 $$\begin{aligned}&\frac{dV}{dt}={div}{\varvec{\tau }} +{\textbf {J}}\times {\textbf {B}}, \end{aligned}$$

3 $$\begin{aligned}&\frac{dT}{dt}=\nabla ^{2}\left( \alpha T\right) +\tau ^{*}\left[ D_{B}\nabla T\cdot \nabla C+\left( \frac{D_{T}}{T_{\infty }}\right) \nabla T\cdot \nabla T\right] -\nabla \left( rq_{r}\right) , \end{aligned}$$

4 $$\begin{aligned}&\frac{dC}{dt}=D_{B}\nabla ^{2}C+\left( \frac{D_{T}}{T_{\infty }}\right) \nabla ^{2}T. \end{aligned}$$

The Cauchy stress tensor of viscosity model, expressing the Williamson fluid is defined as;

5 $$\begin{aligned} {\tau } =-p{\textbf {I}}+\mu {A_{1}}, \end{aligned}$$

where, $\mu$ is kinematic viscosity which is defined below for the Williamson fluid.

6 $$\begin{aligned} \mu =\mu _{\infty }+\frac{\mu _{0}-\mu _{\infty }}{1-\Gamma \gamma ^{.}}, \end{aligned}$$

Here, $\gamma ^{.}$ is shear rate which is defined as

7 $$\begin{aligned} \gamma ^{.}=\sqrt{\frac{1}{2}tr({A_{1}}^{2})}. \end{aligned}$$

In our consideration the term $\mu _{\infty }\ne 0$. So, $\mu$ can be expressed as

8 $$\begin{aligned} \mu =\mu _{0}\left[ \beta ^{*}+\frac{1-\beta ^{*}}{1-\Gamma \gamma ^{.}}\right] , \end{aligned}$$

where the viscosity ratio parameter $B^{*}$ can be defined as;

$$\begin{aligned} \beta ^{*}=\frac{\mu _{\infty }}{\mu _{0}}, \end{aligned}$$

also, the two-dimensional flow’s velocity, temperature and concentration field are

$$\begin{aligned} T=T(r,z,t),C=C(r,z,t),{\textbf {V}}=[u(r,z,t),0,w(r,z,t)]. \end{aligned}$$

The basic equation that deals with mass, momentum, and energy conservation can be stated as follows using the aforementioned geometric model and accompanying assumptions^47^:

**Continuity equation:**

9 $$\begin{aligned} \frac{\partial u}{\partial r}+\frac{u}{r}+\frac{\partial w}{\partial z}=0. \end{aligned}$$

**Momentum equation:**

10 $$\begin{aligned}\frac{\partial w}{\partial t}+u\frac{\partial w}{\partial r}+w\frac{ \partial w}{\partial z}\ &=\frac{\nu }{r}\frac{\partial w}{\partial r}\left[ \beta ^{*}+(1-\beta ^{*})\left( 1-\Gamma \frac{\partial w}{\partial r }\right) ^{-1}\right] -\frac{\sigma H^{2}(t)}{\rho }w \nonumber \\&\quad +\nu \frac{\partial ^{2}w}{\partial r^{2}}\left[ \beta ^{*}+(1-\beta ^{*})\left( 1-\Gamma \frac{\partial w}{\partial r}\right) ^{-1}\right] +\nu \Gamma \frac{\partial w}{\partial r}\frac{\partial ^{2}w}{\partial r^{2} }\left[ (1-\beta ^{*})\left( 1-\Gamma \frac{\partial w}{\partial r} \right) ^{-2}\right] . \end{aligned}$$

**Energy equation:**

11 $$\begin{aligned}\frac{\partial T}{\partial t}+u\frac{\partial T}{\partial r}+w\frac{ \partial T}{\partial z}&=\frac{1}{\rho C_{p}r}\frac{\partial }{\partial r} \left( Kr\frac{\partial T}{\partial r}\right) \nonumber \\&\quad +\tau ^{*}\left[ D_{B}\frac{\partial C}{\partial r}\frac{\partial T}{ \partial r}+\frac{D_{T}}{T_{\infty }}\left( \frac{\partial T}{\partial r} \right) ^{2}\right] -\frac{1}{\rho C_{p}r}\frac{\partial }{\partial r}\left( rq_{r}\right) . \end{aligned}$$

**Concentration equation**

12 $$\begin{aligned} \frac{\partial C}{\partial t}+u\frac{\partial C}{\partial r}+w\frac{\partial C}{\partial z}=D_{B}\left( \frac{\partial ^{2}C}{\partial r^{2}}+\frac{ \partial C}{r\partial r}\right) +\frac{D_{T}}{T_{\infty }}\left( \frac{ \partial ^{2}T}{\partial r^{2}}+\frac{\partial T}{r\partial r}\right) . \end{aligned}$$

In Eq. (11), the terms on left side are due to unsteady flow and convection, while the first term on right side is due to conduction, the next term is because of nanofluid, *K* is variable thermal conductivity and the last term is due to thermal radiation which are defined below^48^. Also, *r* and *z* are, as already mention above, the redial and the axial direction, respectively, and $\nu$ represent kinematic viscosity, $\rho$ is the density of the fluid.

13 $$\begin{aligned} K=K_{\infty }\left( 1+\epsilon \left( \frac{T-T_{\infty }}{T_{f}-T_{0}} \right) \right) ,\quad q_{r}=-\frac{4}{3}\frac{\sigma ^{*}}{k^{*}} \frac{\partial }{\partial r}T^{4}. \end{aligned}$$

In this investigation, the boundary condition are:

14 $$\begin{aligned} \left. \begin{aligned}&u =\frac{U}{\sqrt{1-B t}}, \quad w=\chi \frac{1}{a_{0}^{2}}\frac{4\nu z}{1-B t}+\frac{N_{0}}{\sqrt{1-B t}}\frac{\partial w}{\partial r}, \\&-k\frac{\partial T}{\partial r} =h_{f}(T_{f}-T),\quad C=C_{w}\quad at\quad r=a(t), \\&w \rightarrow 0,\quad T\rightarrow T_{\infty },\quad C\rightarrow C_{\infty }\quad as\quad r\rightarrow \infty . \end{aligned}\right\} \end{aligned}$$

In the above expression, $(\chi >0)$ and $(\chi <0)$ are the representation of the contraction and the expansion of the cylinder respectively.

To find the solution of the governing equations (9)–(12), with associated boundary condition (14), first we will convert them into non-dimensional ordinary differential equations by introducing the following similar variables

15 $$\begin{aligned} \left. \begin{aligned}&w =\frac{1}{a_{0}^{2}}\frac{4vz}{1-Bt}f^{\prime }(\eta ),\ \eta =\left( \frac{r}{a_{0}}\right) ^{2}\frac{1}{1-B t},\ \ \ \phi \left( \eta \right) =\frac{C-C_{\infty }}{C_{w}-C_{\infty }},~ \\&\theta \left( \eta \right) =\frac{T-T_{\infty }}{T_{f}-T_{\infty }}\ ,\ \ u=-\frac{1}{a_{0}}\frac{2v}{\sqrt{1-Bt}}\frac{f(\eta )}{\sqrt{\eta }}. \end{aligned}\right\} \end{aligned}$$

The combined non-dimensional equations are

16 $$\begin{aligned}&\eta f^{\prime \prime \prime }(\eta )+f^{\prime \prime }(\eta )\left[ \beta ^{*}+(1-\beta ^{*})\left( 1-Wef^{\prime \prime }(\eta )\right) ^{-1}\right] +f(\eta )f^{\prime }(\eta )-f^{\prime ^{2}}(\eta ) \nonumber \\&\quad +\frac{\eta }{2}f^{\prime \prime ^{2}}(\eta )\left[ (1-\beta ^{*})\left( 1-Wef^{\prime \prime }(\eta )\right) ^{-2}\right] -A\left( f^{\prime }(\eta )+\eta f^{\prime \prime }(\eta )\right) -Mf^{\prime }(\eta )=0, \end{aligned}$$

17 $$\begin{aligned}&2\eta \theta ^{\prime \prime }\left( 1+\epsilon \theta +\frac{2}{3} Rd\right) +2\theta ^{\prime }+2\epsilon \theta \theta ^{\prime }+\epsilon \theta ^{^{\prime ^{2}}}+\frac{4}{3}Rd\theta ^{\prime } \nonumber \\&\quad +Nb\eta \Pr \left( \phi ^{\prime }\theta ^{\prime }+\frac{Nt}{Nb}\theta ^{\prime ^{2}}\right) +Prf\theta ^{\prime }-PrA\eta \theta ^{\prime }=0, \end{aligned}$$

18 $$\begin{aligned}&\phi ^{\prime }{+\eta }\phi ^{\prime \prime }+Le\left[ \phi ^{\prime }f-A\eta \phi ^{\prime }\right] +\frac{Nt}{Nb}\left( \theta ^{\prime }{+\eta }\theta ^{\prime \prime }\right) =0. \end{aligned}$$

The non-dimensional boundary conditions are described as follows,

19 $$\begin{aligned} \left. \begin{aligned}&f(1) =s\ ,\ f{^{\prime }}(1)=\chi +Nf^{\prime \prime }\left( 1\right) \ ,\ \theta {^{\prime }}(1)=-\gamma (1-\theta (1))\ ,\ \phi (1)=1, \\&f{^{\prime }}(\infty ) \rightarrow 0\ ,\ \theta (\infty )\rightarrow 0\ ,\ \ \phi (\infty )\rightarrow 0. \end{aligned}\right\} \end{aligned}$$

The dimensionless constant *Nt* thermophoresis parameter, *M* magnetic parameter *A* unsteadiness parameter, *Pr* Prandtl number, *s* mass transfer parameter, *Le* Lewis number, $\gamma$ Biot number, *Rd* radiation parameter, *We* local Weissenberg number and *Nb* Brownian motion parameter, $\epsilon$ thermal conductivity parameter, *N* slip parameter, $\chi$ the velocity ratio parameter and these are defined as

20 $$\begin{aligned} \left. \begin{aligned}&We =\frac{8\Gamma rz\nu }{\left( 1-B t\right) ^{2}a_{0}^{4}}\ ,\ M=\frac{\sigma a_{0}^{2}H_{0}^{2}}{4\rho \nu }\ ,\ Nt=\frac{\tau D_{T}(T_{f}-T_{\infty })}{T_{\infty }\nu },\ \gamma =\frac{a_{0}h_{f}\left( 1-B t\right) }{2kr}, \\&Nb =\frac{D_{B}\tau (C_{w}-C_{\infty })}{\nu },\ \Pr =\frac{\nu }{\alpha }\ ,\ Le=\frac{\nu }{D_{B}}\ ,\ s=-\frac{a_{0}U}{2\nu }, \ A=\frac{a_{0}^{2}B }{4\nu }.\\ \end{aligned}\right\} \end{aligned}$$

The significant characteristic of the research is the skin friction coefficient, the Nusselt number and the Sherwood number which are given below

$$\begin{aligned} C_{f}=\frac{\tau _{rz}|_{r=a(t)}}{\frac{1}{2}\rho U^{2}}\ ,\ Nu=\frac{ a(t)q_{w}|_{r=a(t)}}{2k\left( T_{f}-T_{\infty }\right) }\ ,\ Sh=\frac{ a(t)q_{m}|_{r=a(t)}}{2D_{B}(C_{w}-C_{\infty })} \end{aligned}$$

where,

$$\begin{aligned}&\tau _{rz}=\mu _{0}\frac{\partial w}{\partial r} \left[ \beta ^{*}+\left( 1-\beta ^{*}\right) \left( 1-\Gamma \frac{\partial w}{\partial r} \right) ^{-1}\right] , \\&q_{w}|_{r=a(t)}=-K\frac{\partial T}{\partial r}+q_{r},\qquad q_{m}|_{r=a(t)}=-D_{B}\frac{\partial C}{\partial r}, \end{aligned}$$

After using the defined transformation, we obtain

21 $$\begin{aligned} \left. \begin{aligned}&\frac{C_{f}a(t)}{4z} =f^{\prime \prime }\left( 1\right) \left[ \beta ^{*}+\left( 1-\beta ^{*}\right) \left( 1-Wef^{\prime \prime }\left( 1\right) \right) ^{-1}\right] , \\&Nu = -\left( 1+\frac{4}{3}\frac{Rd}{1+\epsilon \theta (1)}\right) \theta ^{\prime }(1). \qquad Sh =-\phi {^{\prime }}(1). \end{aligned}\right\} \end{aligned}$$

## Solution methodology

The governing non linear equations (16)–(18) along with boundary conditions (19) are solved with the help of shooting technique. In shooting technique, we will first convert the higher order system of equation into the first order system of equations. So for conversion, we suppose,

$$\begin{aligned} Let,\ f= & {} h_{1},\ f^{\prime }=h_{2,\ }f^{\prime \prime }=h_{3},\ \theta =h_{4},\ \theta ^{\prime }=h_{5},\ \varphi =h_{6},\ \varphi ^{\prime}=h_{7} \\ \left( \begin{array}{c} h_{1}^{\prime} \\ h_{2}^{\prime} \\ h_{3}^{\prime} \\ h_{4}^{\prime} \\ h_{5}^{\prime} \\ h_{6}^{\prime} \\ h_{7}^{\prime} \end{array} \right)= & {} \left( \begin{array}{c} h_{2}, \\ h_{3}, \\ -h_{3}(\beta^{\ast }+(1-\beta^{\ast })\left( 1-Wef^{\prime \prime }(\eta )\right) ^{-1}) \\ -\frac{We h_{3}^{2}}{2}\left[ (1-\beta^{\ast })\left( 1-Wef^{\prime \prime }(\eta )\right) ^{-2}\right] \\ \frac{-h_{1}h_{3}+h_{2}^{2}+A\left( h_{2}+\eta h_{3}\right) +Mh_{2}} {\eta \left[ (\beta^{\ast }+(1-\beta^{\ast })\left( 1-Weh_{3}\right) ^{-1})+Weh_{3}(1-\beta^{\ast })\left( 1-Weh_{3}\right) ^{-2}\right] }, \\ h_{5}, \\ Pr A\eta h_{5}-\Pr h_{1}h_{5}-2h_{5}-2\epsilon h_{4}h_{5}- \\ \frac{\epsilon h_{5}^{2}-\frac{4}{3}Rdh_{5}-Nb\eta \Pr \left( h_{5}h_{7}+\frac{Nt}{ Nb}h_{5}^{2}\right)} {2\eta \left( 1+\epsilon h_{4}+\frac{2}{3}Rd\right)},\\ h_{7},\\ \frac{-h_{7}-Le\left[ h_{1}h_{7}-A\eta h_{7}\right] -\frac{N_{t}}{N_{b}} \left( h_{5}{+\eta }h_{5}^{^{\prime }}\right) }{\eta }.\\ \end{array}\right) \end{aligned}$$

The above system is numerically integrated through Runge-Kutta method of order 4 within the domain [0,10] after assuming the missing initial conditions at $\alpha _{1}$, $\alpha _{2}$, $\alpha _{3}$. Suppose $h_{3}(1)=\alpha _{1}$, $h_4(1)=\alpha _{2}$, $h_7(1)=\alpha _{3}$. The shooting technique is well-known for its ease of usage and cheap computing cost. In compared to finite difference or any other numerical computational approach, this method is far quicker. Its convergence rate is faster and computational cost is very low. It is effectively used by many authors to tackle the nonlinear differential equations. The results are compared in limiting case and found a good agreement as shown in Table 1.

**Table 1:** Comparison of results in limiting case with Fang et al.^49^ and Hashim et al.^47^, when $s=M=We=\beta ^{*}=0$ for $f^{\prime \prime }(1)$.

*A*	Fang et al.^49^	Hashim et al.^47^	Present
0	− 1.17775	− 1.17776	− 1.177776
− 0.5	− 1.45646	− 1.45643	− 1.456432
− 0.6	–	− 1.55261	− 1.552615
− 0.8	–	− 1.77006	− 1.770063
− 0.9	–	− 1.88949	− 1.889494
− 1.0	− 2.01502	− 2.01503	− 2.015031

## Result and discussion

The behavior of several parameters that are emerging in defined model and their impact on velocity, temperature, and concentration distributions are described graphically. For this purpose, we have drawn various figures (Figs. 2–18). In almost all the figures, we have considered the shrinking cylinder, i.e. $\chi < 0$, unless stated. Following ranges of parameters are considered for the formulation of figures unless specified $0 \le M \le 1.2$, $2 \le s \le 3.5$, $0.2 \le We \le 0.8$, $-0.7 \le A \le -0.1$, $0.1 \le A \le 0.7$, $0.2 \le \gamma \le 0.8$, $0.3 \le Nb \le 0.9$, $2.0 \le Pr \le 5.0$, $0.3 \le Rd \le 0.9$, $0.1 \le Nt \le 0.3$, $0.4 \le Le \le 1.0$.

### Velocity profile behavior

**Figure 2:**
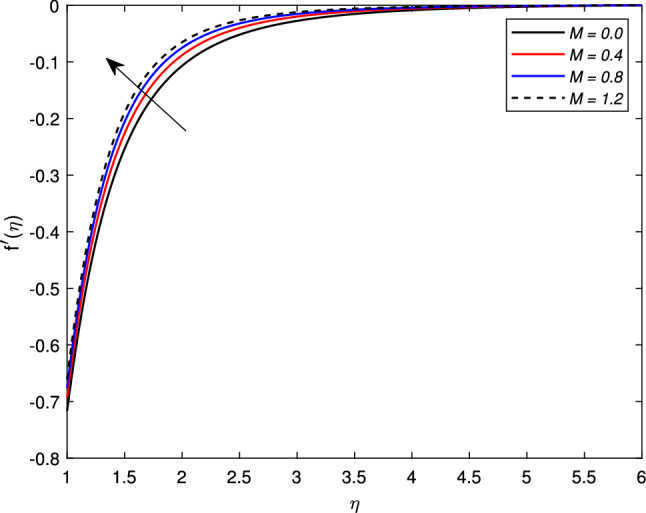
Impact of magnetic parameter *M* on $f^{\prime }(\eta )$ when $A=-0.2$ (contracting cylinder).

**Figure 3:**
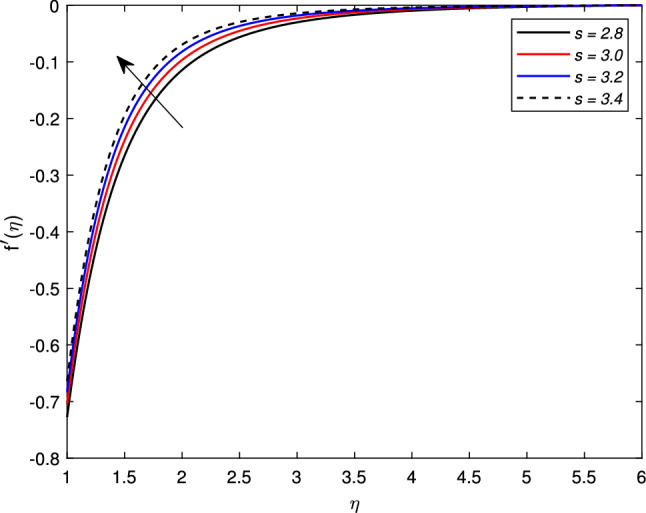
Impact of mass transfer parameter *S* on $f^{\prime }(\eta )$ when $A=-0.2$ (contracting cylinder).

**Figure 4:**
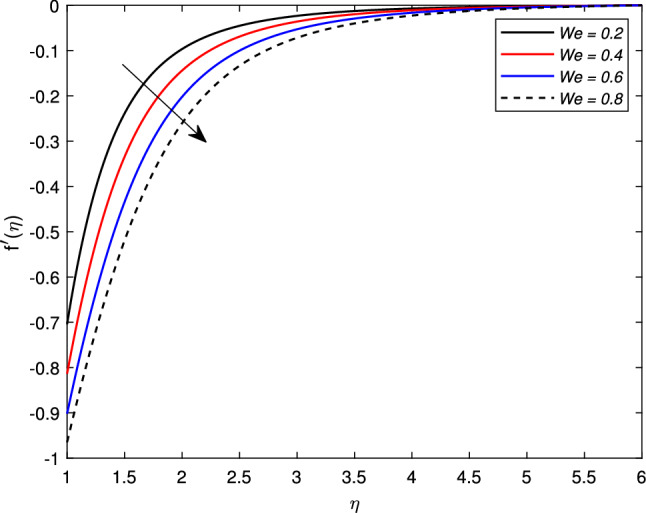
Impact of Weissenbert number *We* on $f^{\prime }(\eta )$ when $A=-0.2$ (contracting cylinder).

**Figure 5:**
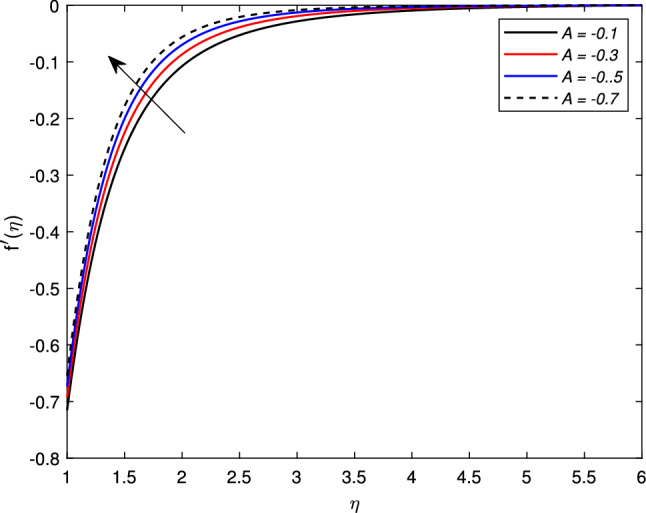
Impact of unsteady parameter *A* on $f^{\prime }(\eta )$ for contracting cylinder.

**Figure 6:**
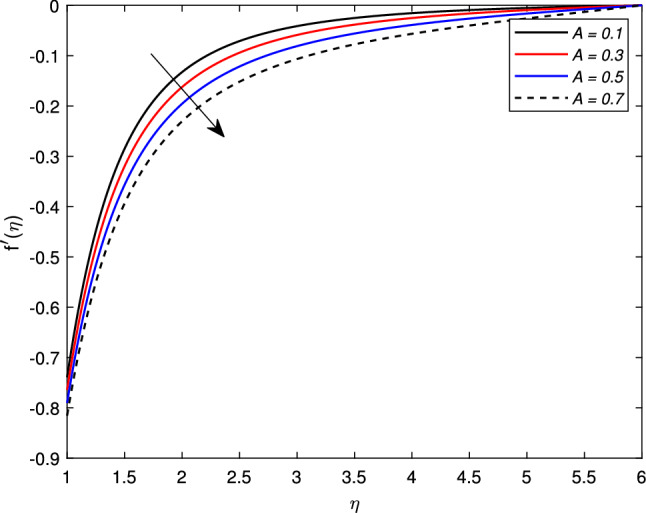
Impact of unsteady parameter *A* on $f^{\prime }(\eta )$ for expanding cylinder.

Figure 2 shows the variation in the velocity profile $f^{\prime }(\eta )$ against the prominent magnetic field *M*. The value of the velocity of the nanofluid increases significantly with the increase in the value of the magnetic parameter *M* for the shrinking case, but there is a decline in the thickness of the momentum boundary layer with the increase in the value of the magnetic field. The Lorentz forces behave unlikely for the shrinking cylinder, so the flow rate increases. Figure 3 shows the effects of mass transfer parameter *S* on the velocity profile $f^{\prime }(\eta )$. This figure shows that the enhancement of the suction parameter grows the speed of the fluid. This figure also demonstrates the shrinking cylinder phenomenon. A dimensionless Weissenberg number *We* is introduced by Karl Weissenberg. Figure 4 portray the velocity profile for the increasing Weissenberg number. Clearly, for the higher Weissenberg number *We*, the velocity profile reduces. This happens as the relaxation time of particles has been extended (particle causes resistance inflow), by maximizing the value of *We*. The unsteadiness parameter *A* showing expansion $A>0$ or contraction $A<0$ of the cylinder has a significant impact on the velocity of the fluid. Figs. 5 and 6 are drawn to see the insight behavior of *A* on flow speed. It has been examined that the unsteadiness parameter *A* for the expanding cylinder $A>0$, rises, the profile of the velocity gets down. But an inverse relation is noted for the contracting cylinder, as the velocity goes up for the numerically higher values of *A*.

### Temperature profile behavior

**Figure 7:**
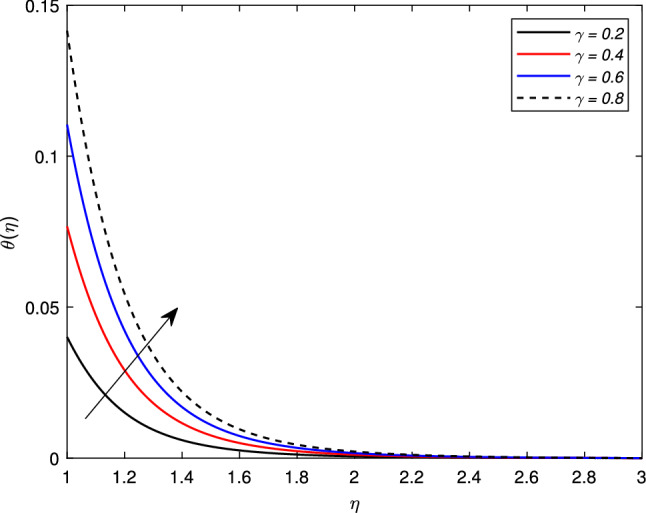
Impact of Biot number $\gamma$ on temperature profile $\theta (\eta )$.

**Figure 8:**
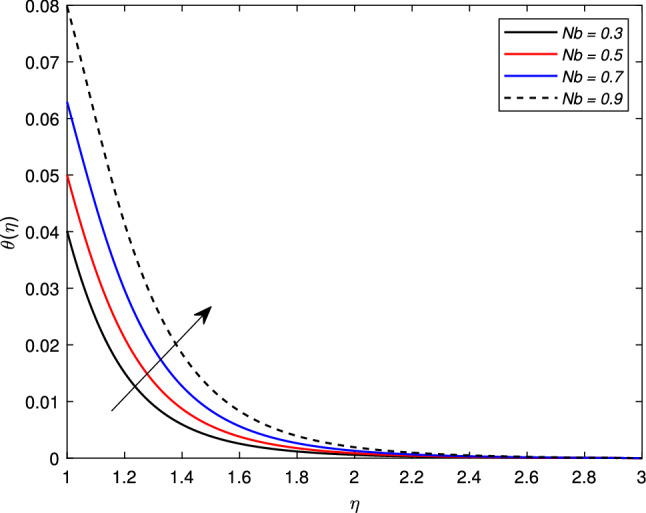
Impact of Brownian motion parameter *Nb* on temperature profile $\theta (\eta )$.

**Figure 9:**
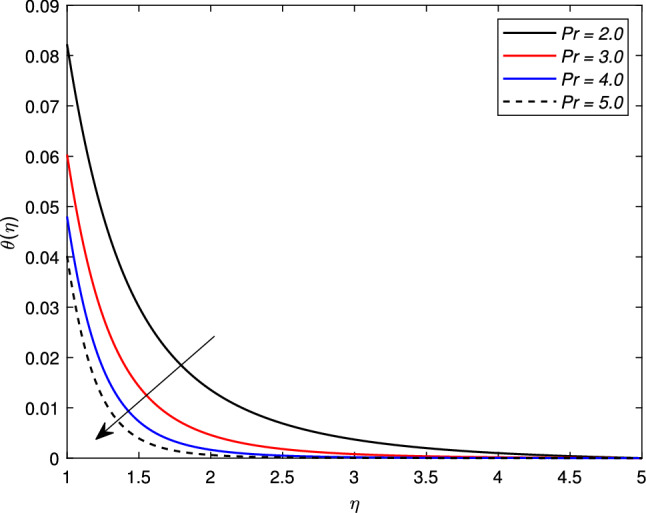
Impact of Prandtl number *Pr* on temperature profile $\theta (\eta )$.

**Figure 10:**
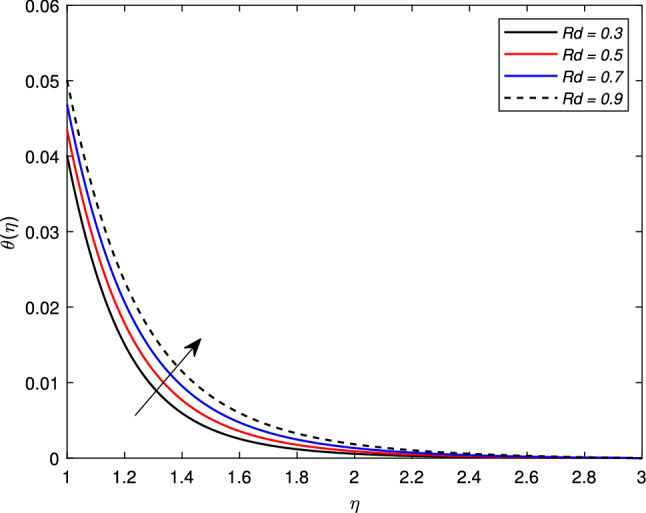
Impact of thermal radiation parameter *Rd* on temperature profile $\theta (\eta )$.

There is an escalation in the thickness of the thermal boundary wall layer for changing the Biot number $\gamma$ against the non-dimensional temperature distribution as depicted in Fig. 7. It is clear from the figure that a rise in the Biot number results an acceleration in the temperature profile. The heat transfer coefficient $h_{f}$ is the main source of this escalation in temperature, as the Biot number is directly related with $h_{f}$. Figure 8 depicts a change in the temperature profile $\theta (\eta )$ for a Brownian motion parameter *Nb*. The non-dimensional parameter *Nb* is directly proportional to the Brownian diffusion coefficient. It is noted that for greater values of *Nb*, the temperature distribution rises. The reason is that as the value of the Brownian parameter accelerates, the collision among fluid particles increases, which ultimately enhance the kinetic energy of molecules, and hence the temperature rises. Figure 9 shows the impact of *Pr* on the temperature distribution. It is observed that by enhancing the *Pr* values, the thermal boundary layer and temperature reduces. Prandtl number is the ratio of the momentum diffusivity to thermal diffusivity. Thermal diffusivity gets highly affected by the Prandtl number. A higher Prandtl number ultimately reduces the thermal conductivity which, as a result, drops the temperature of the nanofluid. The effect of thermal radiation *Rd* on the temperature profile is depicted in Fig. 10. Due to the emission of the electromagnetic radiations, the temperature of the nanofluid improves for the boosted thermal radiation parameter *Rd*.

### Concentration profile behavior

**Figure 11:**
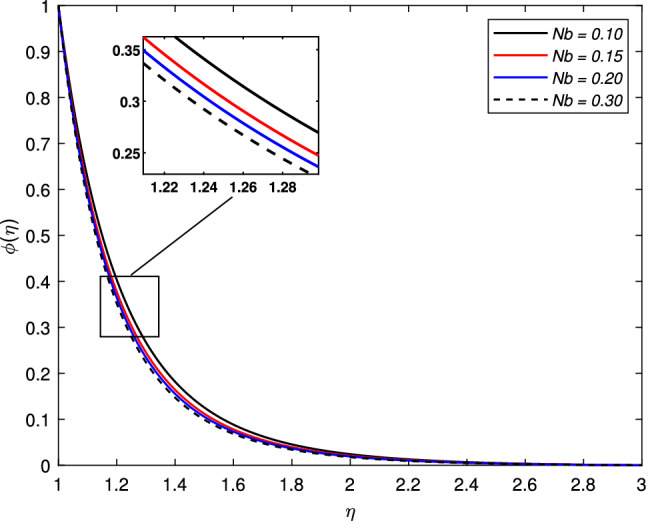
Impact of Brownian motion parameter *Nb* on concentration profile $\phi (\eta )$.

**Figure 12:**
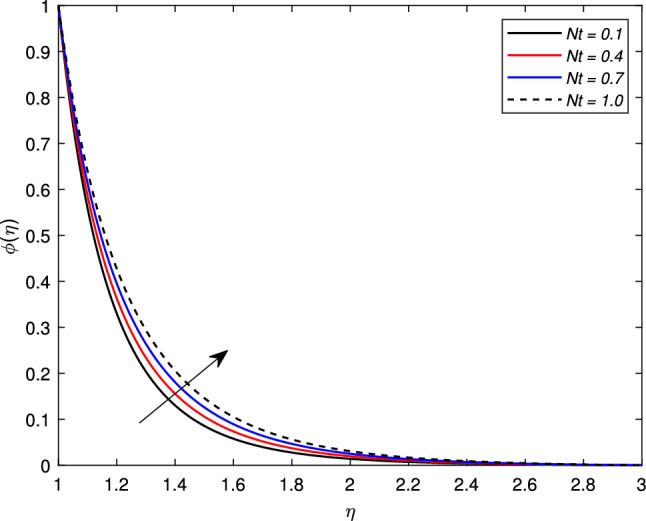
Impact of thermophoresis parameter *Nt* on concentration profile $\phi (\eta )$.

**Figure 13:**
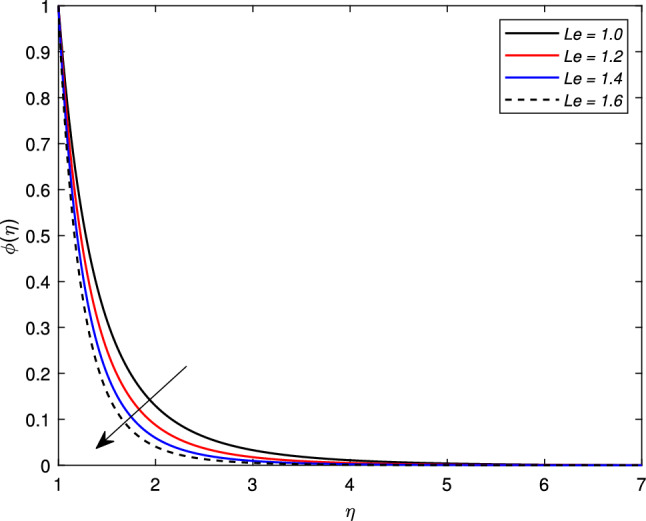
Impact of Lewis number *Le* on concentration profile $\phi (\eta )$.

The influence of Brownian motion parameter *Nb* on the concentration profile is highlighted in Fig. 11. The concentration steadily decreases with higher Brownian motion *Nb*. The reason is that the greater values of the Brownian parameter accelerates collision among fluid particles and reduces the viscosity of the nanofluid. In the next Fig. 12, the consequence of thermophoresis parameter *Nt* on the concentration of the nanofluid $\phi (\eta )$ is displayed. It is noted through the graph that the concentration profile increases when the values of *Nt* are enhanced. It is due to the fact that the thermo-diffusion coefficient *Dt* is directly linked with the thermophoresis parameter. Higher values of *Nt* imply the increment in the diffusion coefficient which escalates the concentration profile. Lewis number which is the ratio of viscosity to Brownian diffusion coefficient has an inverse relation with the concentration distribution. As observed in Fig. 13, the concentration of the nanofluid reduces significantly for the escalating values of *Le*.

### Skin friction, Nusselt number and Sherwood number

**Figure 14:**
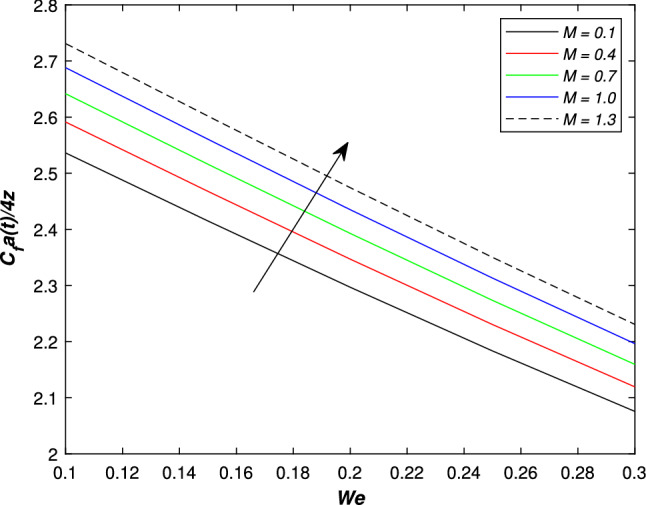
Impact of magnetic parameter *M* and Weissenber number *We* on skin friction coefficient.

**Figure 15:**
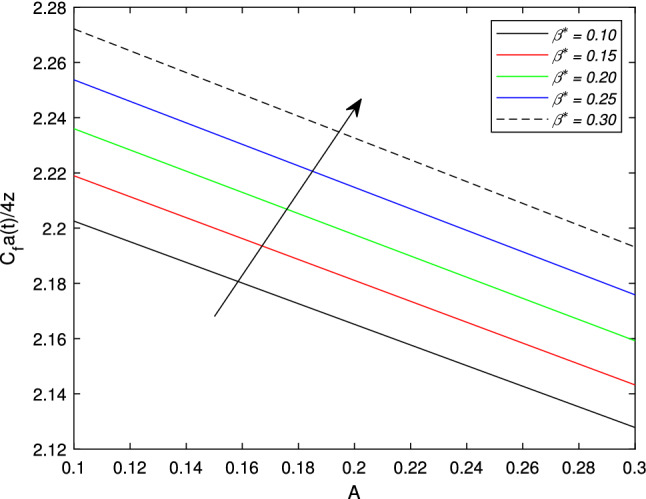
Impact of unsteadiness number *A* and viscosity ratio parameter $\beta ^{*}$ on skin friction coefficient.

**Figure 16:**
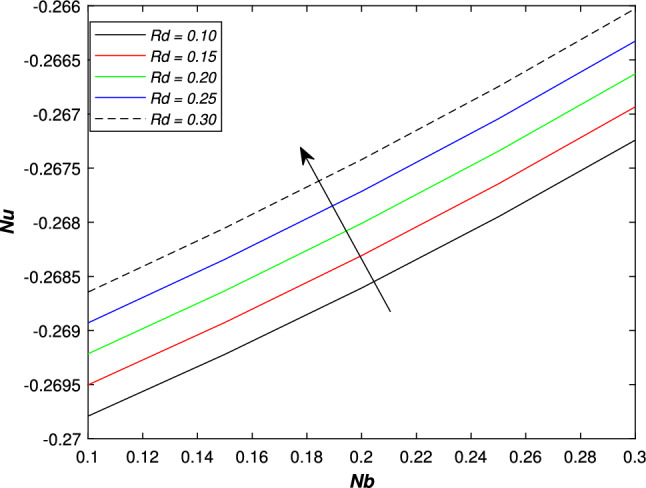
Impact of Brownian motion parameter *Nb* and thermal radiation parameter *Rd* on Nusselt number.

**Figure 17:**
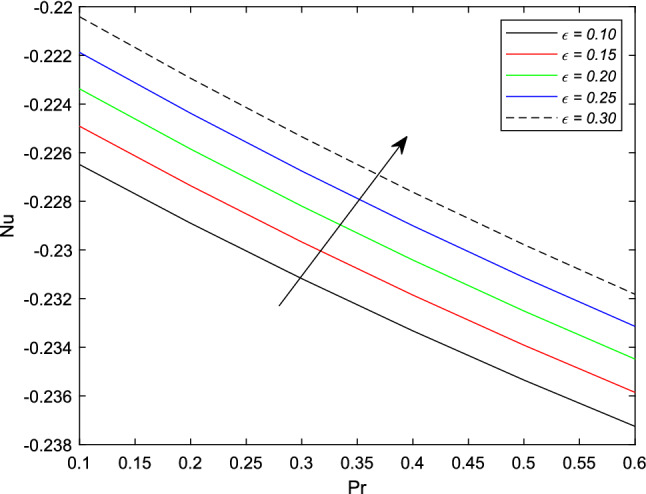
Impact of thermal conductivity parameter $\epsilon$ and Prandtl number *Pr* on Sherwood number.

**Figure 18:**
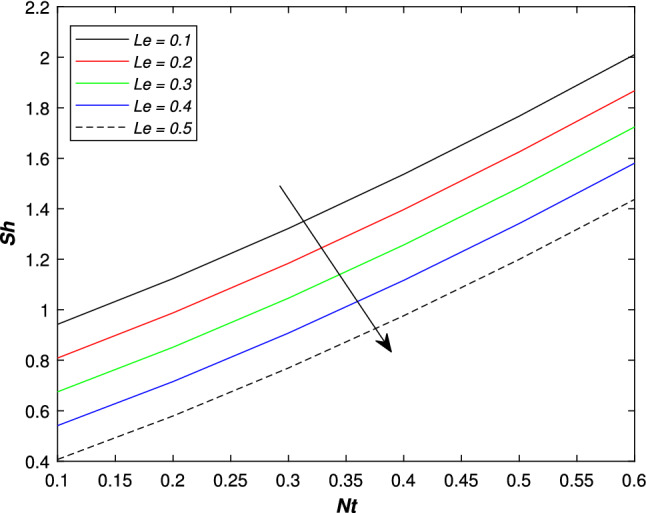
Impact of Lewis number *Le* and thermophoresis parameter *Nt* on sherwood number.

In the upcoming figures, the influence of different parameters on the Skin friction, Nusselt, and Sherwood number is shown. In Fig. 14, it is observed that the mounting values of magnetic parameter *M* can rise the numerical values of skin friction coefficient but on the other hand, the Weissenberg number shows an opposite relation. Skin fraction gets down for the growing values of *We*. The influence of viscosity ratio parameter $\beta ^{*}$ and unsteadiness parameter *A* on the skin friction coefficient is displayed in Fig. 15. While increasing the viscosity ratio parameter $\beta ^{*}$, an escalating trend in the non-dimensional skin friction coefficient is observed. The unsteadiness parameter *A* has an inverse relation with the drag coefficient. To see the influence of Brownian motion parameter *Nb*, thermal conductivity parameter $\epsilon$, Prandtl number *Pr* and thermal radiation parameter *Rd* on the heat transfer rate, Figs. 16 and 17 are drawn. Analysis reveals that higher Brownian motion, thermal conductivity, and thermal radiation parameter results in an uprising drift in the rate of heat transfer. Prandtl number shows a decline curve for the Nusselt number. In the last Fig. 18, Lewis number *Le* shows a decreasing behavior against the mass transfer rate on the surface of the shrinking cylinder. The thermophoresis parameter *Nt* behaves to increase the Sherwood number.

## Conclusion

This research looks at the outcomes of transient Williamson fluid flow as well as heat transmission in the presence of nanoparticles. A contracting and expanding cylinder is employed to move the fluid, while the convective boundary wall state at the surface is taken into account. Buongiorno’s model was used to analyze the impact of nanofluid flow. The thermal radiation effect and slip condition also applied on cylinder.

- Magnetic parameter has an increasing trend in velocity profile for the shrinking case.
- Weissenberg number shows a decline variation for the velocity field.
- The temperature field grows for the Biot number due to heat transfer coefficient.
- The unsteadiness parameter for the expanding cylinder uplifts the flow rate.
- Skin friction increases as the higher unsteadiness parameter is increased for the lower solution.

This research work can be extended by incorporating Joule heating, viscous dissipation, activation energy, chemical reaction impacts in energy or concentration equation.

## Data Availability

The datasets used and/or analysed during the current study available from the corresponding author on reasonable request.

## List of symbols

*We*: Weissenberg number
*Nt*: Thermophoresis parameter
*Pr*: Prandtl number
*s*: Mass transfer parameter
*A*: Unsteady parameter
$\chi$: Stretching/shrinking parameter
*Nu*: Nusselt number
*Rd*: Thermal radiation
*N*: Slip condition
$$\phi$$: Dimensionless concentration
$\sigma ^{*}$: Stephan Boltzman parameter
$$(u,w) \,\mathrm{(m/s)}$$: Velocity components
$$\beta$$: Dimensional constant
$$\mu \,\mathrm{(kg/ms)}$$: Kinematic viscosity
$$\sigma \,\mathrm{(simens/m)}$$: Electric conductivity
$$\eta$$: Similarity variable
$$\alpha \,(\mathrm{m}^{2}/\mathrm{s})$$: Thermal diffusivity
*U*: Mass transfer coefficient
$\Gamma$: Time rate constant
$$C \,(\mathrm{mol/m}^{3})$$: Concentration
*M*: Magnetic parameter
*Nb*: Brownian motion parameter
*Le*: Lewis number
$$\gamma$$: Biot number
$\beta ^{*}$: Viscosity ratio parameter
*Sh*: Sherwood number
$K\,\mathrm{(W/mK)}$: Variable thermal conductivity
$q_{r}\,(\mathrm{kg/ms}^{3}\,\mathrm{K})$: Thermal radiation coefficient
$$\theta$$: Dimensionless temperature
$$v\,(\mathrm{m}^{2}/\mathrm{s})$$: Dynamic velocity
$k^{*}$: Mean absorption coefficient
$(r,z)\,\mathrm{(m)}$: Cylindrical coordinate system
$\epsilon$: Thermal conductivity parameter
$C_{f}$: Skin friction coefficient
$$\rho \,(\mathrm{kg/m}^{3})$$: Density
*H*(*tesla*): Magnetic field strength
$T \,(\mathrm{K}))$: Temperature of fluid
$$p\,(\mathrm{Pa})$$: Pressure
$D_{B}\,(\mathrm{m}^{2}/\mathrm{s})$: Brownian diffusion coefficient
$D_{T}\,(\mathrm{m}^{2}/\mathrm{s})$: Thermophoretic diffusion coefficient
